# Prospects for Trace Analysis in the Analytical Electron Microscope

**DOI:** 10.6028/jres.093.081

**Published:** 1988-06-01

**Authors:** D. B. Williams

**Affiliations:** Department of Materials Science and Engineering, Lehigh University, Bethlehem, PA 18015

## Background

**Figure 1:**
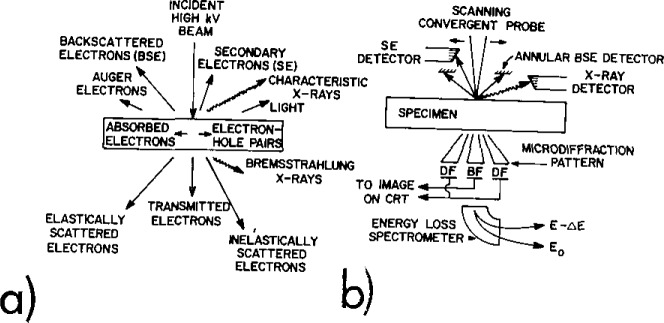
a. Schematic diagram showing the range of signals generated when a high kV electron beam strikes a thin foil sample. b. Typical array of detectors in a modern analytical electron microscope.

The analytical electron microscope (AEM) uses a high energy (≥100 kV) beam of electrons to generate a range of signals from a thin foil sample as shown in figure 1a [1,2]. Various detectors are configured in the AEM to pick up most of the generated signals (fig. 1b). Microanalysis is usually performed using the characteristic x-ray signal, detected by an energy dispersive spectrometer (EDS) although occasionally the electron energy loss spectrum is also used. This paper will emphasize x-ray microanalysis only. The specific advantages that the AEM has for microanalysis are two-fold. First the instrument can be operated as a high resolution transmission electron microscope, thus permitting the analytical information to be related directly to the microstructure of the sample. Second, in the AEM most microanalysis is performed with a probe size < ≈ 10 nm and a specimen thickness < ≈ 100 nm. This results in an analyzed volume ≈ 10^−5^ of that commonly encountered in bulk microanalysis, for example, in the electron probe microanalyzer (EPMA). This small volume means that the spatial resolution of microanalysis is relatively good (routinely <50 nm) but generally trace analysis in the AEM is relatively difficult, because generated signal intensities are low.

## X-Ray Microanalysis in the AEM

The definition of “trace analysis” in this paper is assumed to be that commonly used in the EPMA, namely elemental concentrations < ≈ 0.5 wt% [3]. Under these conditions the average counts in the x-ray characteristic peak, $\bar{N}$, approach the average counts in the background, ${\bar{N}}^{b}$.

One reasonable measure of analytical sensitivity used in the AEM field is the minimum mass fraction of one element that is detectable in the matrix of another. Using the criterion of Liebhafsky et al. [4], the peak is detectable if:

$$\bar{N}>3{\left(2{\bar{N}}^{b}\right)}^{1/2}$$ (1)

This simple criterion can be combined with the Cliff-Lorimer equation [5] to give a minimum mass fraction of element B (*C*_B_):

$$C_{B}={\frac{3(2I_{B}^{b})}{I_{A}-I_{A}^{b}}}^{1/2}\cdot C_{A}\cdot k_{\mathrm{AB}}-1$$ (2)

where $I_{A}^{b}$ and $I_{B}^{b}$ are background intensities for elements A and B; *I*_A_ is the integrated characteristic intensity from A; *C*_A_ is the concentration of A (in wt%) and *k*_AB_^−1^ is the reciprocal of the Cliff-Lorimer sensitivity *k*-factor *k*_AB_ [5]. The equation can be rewritten [6] as:

$$C_{B}={\frac{3(2I_{B}^{b})}{I_{B}-I_{B}^{b}}}^{1/2}\cdot C_{B}$$ (3)

Results using eqs (2) and (3) have been given by Romig and Goldstein [7] (≈ 0.5% Ni in Fe), Michael [6] (≈0.07% Mn in Cu) and Lyman [8] (≈0.1% Ni in Fe). The results of Michael [6] are shown in table 1. What is not apparent in these reported values is that since all the data were obtained from homogeneous samples, spatial resolution was of little consequence and was usually >50 nm which is the current limit for most thermionic source AEMs. The data in table 2 [9] are the first to compare the effect of spatial resolution on minimum detectability. These results show that a sensitivity <0.1 wt% Cr with moderate spatial resolution (< ≈ 50 nm) can only be achieved with an AEM employing a field emission gun, such as the Vacuum Generators HB501. Thermionic source instruments such as the Philips EM430 can only demonstrate <0.1 wt% detectability with substantially poorer spatial resolution.

## Future Prospects for X-Ray Analysis in the AEM

However, recent instrumental developments promise substantial improvement in trace analysis capability in the AEM. A combination of higher voltage beams (up to 400 kV), brighter (field emission) electron sources, improved microscope stage design [10] and x-ray spectrometry advances offer the prospect of extending the minimum mass fraction detectable by x-ray analysis down to ≈0.01 wt% [8], If this can be achieved while maintaining spatial resolution at the 10 nm level or below, then the AEM will be close to detecting the presence of only a few atoms, as well as localizing them to within a few tens of unit cells.

From an experimental standpoint, Ziebold [11] has shown that *C*_B_ depends on several factors, namely:

$$C_{B}\propto {\left(I_{B}\cdot I_{B}/I_{B}^{b}\cdot \tau \right)}^{-1/2}$$ (4)

where τ is the counting time to acquire the peak. Going to an intermediate voltage such as 300 kV, will increase the value of *I*_B_ (the peak intensity) and $I_{B}/I_{B}^{b}$ (the peak to background ratio (*P/B*)) [8]. Unfortunately, there is no generally accepted definition of *P/B.* A recent attempt has been made to generate a “standard” sample from which to measure a “standard” *P/B* [12,13].

The standard sample is a 100 nm of evaporated Cr on a carbon film, supported on a Cu grid, and manufactured at the National Bureau of Standards.^1^ The value of the *P/B* used is that originally suggested by Fiori et al. [14] and ratios the intensity in the full peak to the average background in a 10 eV channel. Thus the ratio is defined as *P/B* (10 eV).

Preliminary results (table 3) [13] indicate that modern AEMs show an enormous range in *P/B* (10 eV) at 100 kV and not all intermediate voltage instruments show the expected improvement at higher kVs. Nevertheless, an improved *M*_DL_ of ≈0.05 wt% in a 10 nm probe is estimated at 300 kV. However, if an FEG were added to a 300 kV AEM, a probe current of 5 × 10^−8^ to 10^−7^ A should be available in a 10 nm probe. This increase in probe current would result in an increase in P of 100 times and would improve the *M*_DL_ by ≈10 times to 0.01 wt% in a nominal 100 to 200 nm thick film at 300 kV [8]. Such an improvement of over an order of magnitude in analytical sensitivity brings x-ray analysis in the AEM into the 100 ppm range similar to that obtained in the electron probe microanalyzer. None of these calculations takes into account the possibility of increasing the value of τ in eq (4). Typically τ is limited by contamination, specimen drift and operator fatigue, Contamination can be virtually eliminated by careful specimen preparation and good (<10^−8^ Torr) vacuums. Specimen drift can now be compensated electronically [15], effectively eliminating operator fatigue and permitting such experiments as overnight counting, long-term digital mapping and other techniques, hitherto the realm of classical bulk analysis using the EPMA at the micron level.

## Figures and Tables

**Table 1:** Calculation of the minimum mass fraction of Mn detectable in Cu using eq (3)

*I*_Mn_	$I_{\mathrm{Mn}}^{b}$	$\left(I_{\mathrm{Mn}}-I_{\mathrm{Mn}}^{b}\right)$	3(2*I*^b^)	$C_{\mathrm{Mn}}(\mathrm{MMF})=C_{\mathrm{mn}}\times \frac{3(2I^{b})}{\left(I_{\mathrm{Mn}}-I_{\mathrm{Mn}}^{b}\right)}\;\mathrm{wt}\%$
11904	1995±189	9909±422	189±9	0.064±0.008
13769	2299±203	11470±454	203±9	0.059+0.007
10737	1860±183	8877±400	183±9	0.069±0.008
10547	1916±186	8631±394	186±9	0.072±0.009
				av. = 0.067±0.008 wt%

Specimen Cu 3.36 wt% Mn.

Data obtained at 120 kV, 20 nm probe size, 40 μA emission current, 70 μm C_2_ aperture, W hairpin filament.

From refs [1,7]. Reproduced courtesy of Philips Electronic Instruments Publishing Group.

**Table 2:** Calculated MMF values for Cr after 200 s livetime and spatial resolution of microanalysis for a range of AEMs

Microscope	Probe current (nA)	Accelerating voltage (kV)	Sample Thickness (nm)	Cr MMF wt%	Calculated spatial resolution (nm)
HB-501	0.5	100	164	0.125	45
HB-501	1.7	100	164	0.069	45
HB-501	0.5	100	434	0.056	200
HB-501	1.7	100	434	0.035	200
EM430	0.5	100	164	0.181	70
EM430	0.8	300	164	0.135	25
EM430	0.5	100	434	0.054	200
EM430	0.8	300	434	0.053	70

Data from ref. [8]. Reproduced by permission of C. E. Lyman and San Francisco Press.

**Table 3:** Peak to background (*P/B* (10 eV)) data for the CrK_a_ peak obtained from a standard thin film sample in a range of AEMs

AEM	kV	α^°^	Ω(sr)	*P/B*
1	120	20	0.13	1621
2	100	20	0.13	3346
2	200	20	0.13	3181
2	300	20	0.13	2991
3	300	25	0.13	2983
4	100	20	0.13	3177
5	200	72	0.03	2489
6	200	72	0.01	2873
7	100	10	0.02	3007
7	100	13	0.077	2690
8	100	13	0.077	3120
9	120	20	0.13	2879
10	100	30	0.13	2255
11	100	25	0.04	3040
12	200	34	0.005	2300
12	200	37	–	3300
13	120	20	0.13	3093

Reproduced courtesy of San Francisco Press.

α^°^ = detector take-off angle above horizontal.

Ω = delector solid angle.

## Footnotes

1 Standard films may be obtained From Dr. E, B. Steel, Analytical Chemistry Division, Bldg. 222, Room A121, National Bureau of Standards, Gaithersburg, MD 20899.
